# Brain-specific *Crmp*2 deletion leads to neuronal development deficits and behavioural impairments in mice

**DOI:** 10.1038/ncomms11773

**Published:** 2016-06-01

**Authors:** Hongsheng Zhang, Eunchai Kang, Yaqing Wang, Chaojuan Yang, Hui Yu, Qin Wang, Zheyu Chen, Chen Zhang, Kimberly M. Christian, Hongjun Song, Guo-li Ming, Zhiheng Xu

**Affiliations:** 1 State Key Laboratory of Molecular Developmental Biology, Institute of Genetics and Developmental Biology, Chinese Academy of Sciences, Beijing 100101, China; 2 Institute for Cell Engineering, Johns Hopkins University School of Medicine, Baltimore, Maryland 21205, USA; 3 Department of Neurology, Johns Hopkins University School of Medicine, Baltimore, Maryland 21205, USA; 4 State Key Laboratory of Biomembrane and Membrane Biotechnology, College of Life Sciences, Peking University, Beijing 100871, China; 5 Department of Neurobiology, Shandong Provincial Key Laboratory of Mental Disorders, School of Medicine, Shandong University, Jinan, Shandong 250012, China; 6 The Solomon H. Snyder Department of Neuroscience, Johns Hopkins University School of Medicine, Baltimore, Maryland 21205, USA; 7 Department of Psychiatry and Behavioral Science, Johns Hopkins University School of Medicine, Baltimore, Maryland 21205, USA; 8 Translational Medical Center for Stem Cell Therapy, Shanghai East Hospital, Tongji University School of Medicine, Shanghai 200120, China; 9 Parkinson’s Disease Center, Beijing Institute for Brain Disorders, Beijing 100871, China

## Abstract

Several genome- and proteome-wide studies have associated transcription and translation changes of *CRMP2* (collapsing response mediator protein 2) with psychiatric disorders, yet little is known about its function in the developing or adult mammalian brain *in vivo*. Here we show that brain-specific *Crmp2* knockout (cKO) mice display molecular, cellular, structural and behavioural deficits, many of which are reminiscent of neural features and symptoms associated with schizophrenia. cKO mice exhibit enlarged ventricles and impaired social behaviour, locomotor activity, and learning and memory. Loss of *Crmp2* in the hippocampus leads to reduced long-term potentiation, abnormal NMDA receptor composition, aberrant dendrite development and defective synapse formation in CA1 neurons. Furthermore, knockdown of *crmp2* specifically in newborn neurons results in stage-dependent defects in their development during adult hippocampal neurogenesis. Our findings reveal a critical role for CRMP2 in neuronal plasticity, neural function and behavioural modulation in mice.

## Abstract (web summary)

The *in vivo* function of CRMP2 is unclear. Zhang *et al*. generate and characterize brain-specific *Crmp2* knockout mice. These mice show impairments in hippocampal neurogenesis, neuronal maturation and synaptic transmission, and exhibit schizophrenia-related behavioral deficits.

## Graphical abstract

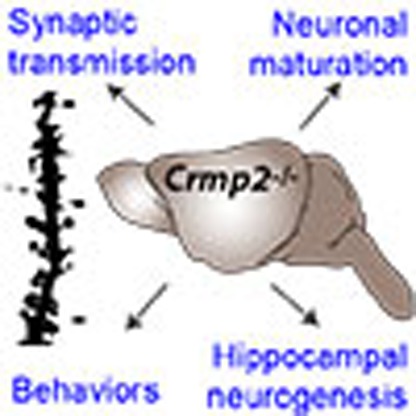

Dysregulated neural development can lead to chronic impairments in neural function and increased vulnerability to environmental toxins or stressors. Among the many factors that can disrupt early brain development, there are a few known genetic mutations that are causal for specific disorders, and many more that have been implicated to varying degrees in conferring risk for psychiatric disorders with a developmental origin. Schizophrenia is one such psychiatric disorder that affects ∼1% of the general population^1^,^2^,^3^. Expression and severity of symptoms can be heterogeneous but several clusters of symptoms are considered to be hallmarks of the disease, including positive symptoms (such as delusions, hallucinations, disordered thoughts and abnormal motor behaviour), negative symptoms (decreased motivation, reduced expressivity and anhedonia) and cognitive deficits in attention, learning and memory^4^,^5^. Pathology identified from imaging and post-mortem analyses includes reduced hippocampal and cortical volume^4^, enlarged lateral ventricles^6^, decreased neuronal density and size^7^,^8^ and reduced dendritic arborization and spine density^9^. Schizophrenia is a highly heritable disorder and dysregulated neurodevelopmental processes are thought to play a key role^2^,^3^. Major advancements have recently been made in identification of genetic risk factors for schizophrenia^10^, and understanding biological roles of these risk factors in brain circuitry formation and maintenance at the molecular, cellular and behavioural levels can lead to a better picture of disease aetiology and targeted strategies for future drug development.

Several genetic association and linkage studies have implicated chromosome region 8p21 in conferring susceptibility for schizophrenia^11^,^12^,^13^. Subsequent linkage studies and a linkage meta-analysis suggested associations of *CRMP2* (collapsing response mediator protein 2) located at 8p21.2 with schizophrenia in large family samples from various ethnic cohorts^14^,^15^. Furthermore, functional genetic variants of *CRMP2* have been suggested to be associated with both schizophrenia risk and lower expression of CRMP2 in post-mortem brains of schizophrenia patients^16^. Corroborating these genetic studies, proteome-wide analyses have found significantly altered CRMP2 protein expression levels in post-mortem brains of schizophrenia patients^17^,^18^,^19^,^20^. CRMP2, a microtubule-associated protein, belongs to the CRMP family with 5 homologues (CRMP1–5). Knockout studies of all other CRMPs, except CRMP2, have been reported^20^. *In vitro*, CRMP2 has been shown to regulate various aspects of neuronal development, including axonal and dendritic growth^21^, synaptic physiology and neurotransmitter release^22^,^23^. Knockdown experiments in the embryonic mouse brain further suggest a role of CRMP2 in neuronal polarity and migration^24^,^25^. In addition, constitutive activation of CRMP2 through *Crmp2 S522A* knockin mice exhibit deficits in cortical dendrite patterning in the adult brain^26^,^27^. The functional role of CRMP2 in neuronal plasticity and animal behaviour *in vivo*, however, remains unknown.

Here we report that brain-specific *Crmp2* knockout (cKO) mice exhibit increased locomotion, and social, cognitive and affective behavioural impairments. At the molecular and cellular levels, these cKO mice display aberrant composition of NMDA receptor subunits and abnormal long-term potentiation of synaptic transmission. Furthermore, knockdown of *Crmp2* in newborn neurons in the adult mouse dentate gyrus results in deficits in dendritic and synaptic formation at specific time points of the neuronal developmental process. Our study provides strong evidence at multiple levels to support an important role of CRMP2 in neural development, circuitry integrity and brain function. Furthermore, our results provide mechanistic insight on how mutations in this gene may contribute to developmental and behavioural phenotypes.

## Results

### Generation of *Crmp2* brain-specific KO mice

**Figure 1:**
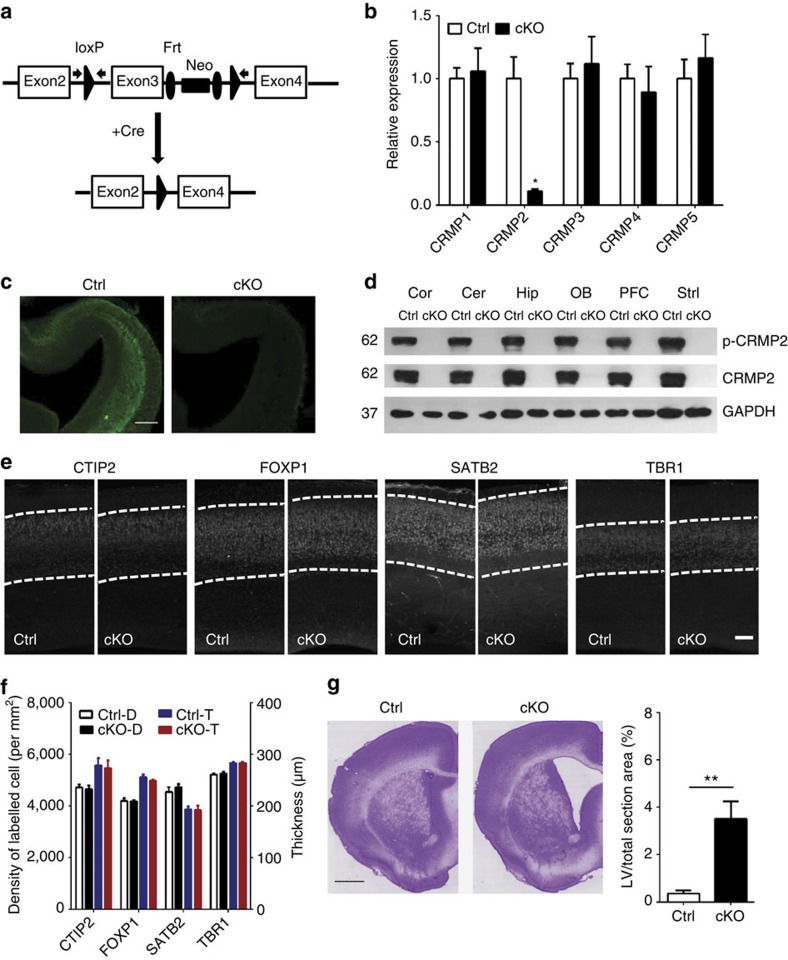
Brain-specific knockout of *Crmp2* leads to enlarged lateral ventricles in mice. (**a**) Strategy for the generation of conditional *Crmp2* knockout mice. (**b**) Crmp2 mRNA levels are significantly lower in cKOs while other *Crmp* family members are not affected as assessed by quantitative real time PCR. *Crmp* mRNA levels in cKOs are normalized to that of Ctrls. Values represent mean±s.e.m. (Ctrl: *n*=4, cKO: *n*=4; **P<0.01; one-way ANOVA with Tukey *post hoc* test). (**c**) Representative brain sections from control (Ctrl) and brain-specific *Crmp2* knockout (cKO) mice stained with CRMP2 antibody. Scale bar, 50 μm. (**d**) CRMP2 and p-CRMP2 are not present in different brain regions of cKOs. Shown are sample western blot analysis. Thirty micrograms of protein was loaded in each lane with GAPDH used as loading control. (**e**) Grossly normal cortical lamination at E18.5 as shown by layer-specific markers, including CTIP2, SATB2, TBR1 and FOXP1. Scale bar, 50 μm. (**f**) Summary of the thickness and density of cells with different cortical layer markers. Values represent mean±s.e.m. (*n*=4 animals for each condition). (**g**) Representative images of coronal sections from p56 brains (Nissl staining), cKOs show enlarged lateral ventricles (LV). Scale bar, 1 mm. Right panel: quantitative analysis of LV from six cKOs and their wild-type littermates (Ctrls). Values represent mean±s.e.m. (Ctrl: *n*=6, cKO: *n*=6; **P<0.01; ANOVA).

CRMP2 is broadly and highly expressed in multiple brain areas, including cortex, hippocampus and cerebellum, both during early postnatal development and in the adult brain^20^. To investigate the impact of CRMP2 expression during neural development, we generated a *Crmp2* conditional null mutant through targeted deletion of exon 3, which causes a frame shift (Fig. 1a; Supplementary Fig. 1a). To specifically disrupt the *Crmp2* gene in the nervous system, these mice were crossed with *Nestin-Cre* mice to generate *Crmp2* cKO. *Crmp2* deletion in different brain areas in cKO mice was confirmed at both protein and mRNA levels (Fig. 1b–d). We also analysed mRNA levels of other *Crmp* family members and did not find an apparent compensatory effect due to *Crmp2* deletion (Fig. 1b). cKO brains exhibited largely normal surface features and cortical lamination with comparable density and thickness of both deep layer and upper layer cortical cells labelled by CTIP2, FOXP1, SATB2 and TBR1 (Fig. 1e,f; Supplementary Fig. 1c,d). Interestingly, most cKO mice displayed a variable degree of lateral ventricle enlargement (Fig. 1g), similar to what has been observed in imaging studies of schizophrenia patients^6^, in addition to lower overall body weight (Supplementary Fig. 1b).

### Behavioural deficits by *Crmp2* cKO mice

**Figure 2:**
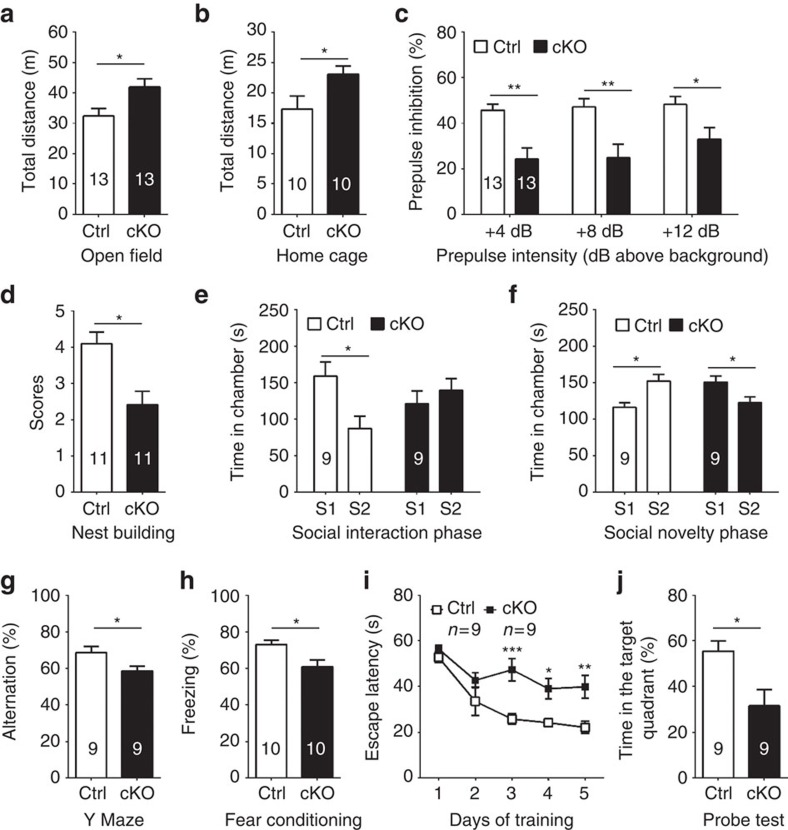
Brain-specific *Crmp*2 knockout mice display schizophrenia-related behaviours. (**a**–**c**) Summary plots of total distance in the open-field test (**a**), home-cage activity (**b**) and prepulse inhibition (**c**). (**d**–**f**) cKOs display social withdrawal as shown by lower nest scores in the nest building test (**d**), less time spent in the chamber containing the social partner (S1) and more time in the chamber containing the empty cage (E) in the social interaction test (**e**), and reduced preference for the novel social partner (S2) and increased time spent in the chamber with familiar social partner S1 in the social novelty test (**f**). (**g**–**j**) cKOs display impaired hippocampal-dependent learning and memory deficits as shown in the Y maze test (**g**) in the fear conditioning test (**h**) and in the Morris water maze test (**j**). Values represent mean±s.e.m. (*P<0.05, **P<0.01, ***P<0.001; **c**,**e**,**f**,**i**: one-way ANOVA with Tukey *post hoc* test; all others: unpaired *t*-test). Numbers of animals used in each test are indicated in each bar graph or as indicated.

To determine whether *Crmp2* cKO mice express behavioural impairments, we subjected these animals to a battery of behavioural tests. In the open-field test, cKO mice showed increased locomotion compared with their wild-type littermates (Ctrl, hereafter; Fig. 2a). Although hyperactivity has been associated with animal models of various disorders, it has been proposed that this phenotype may be analogous to psychomotor agitation in schizophrenia patients^28^,^29^. Moreover, the hyperactivity was suppressed by administration of an antipsychotic drug, clozapine (Supplementary Fig. 2a). cKO mice were also hyperactive in their home cages (Fig. 2b), indicating that their open-field behaviour was not simply a reaction to a novel environment. In addition, there was no significant difference between cKO mice and Ctrls in the time spent in the centre of the open-field (Supplementary Fig. 2b), or the fraction of time spent in the open or closed arms of the elevated plus maze (Supplementary Fig. 2c). These results suggest that increased locomotor activity is not due to altered anxiety levels. Motor coordination, balance and motor learning skills appeared to be intact as measured by performance on an accelerating rotarod test (Supplementary Fig. 2d)

Prepulse inhibition (PPI) is a common measure for assessing sensorimotor gating, which is impaired in many schizophrenic patients and in animals models of psychosis^30^,^31^. We found that cKO mice displayed a significant PPI deficit at multiple intensities of prepulse stimuli (Fig. 2c). Importantly, there was no significant difference in the startle response between cKOs and Ctrls, suggesting no apparent hearing deficit (Supplementary Fig. 2e).

Social behaviour in animals can be quantified in several assays to evaluate interactions with conspecifics^32^. Nest building is often used to assess well-being, social and mating behaviours. We found that cKO mice had a substantially decreased nesting score measured after 16 h with the nestlet material (Fig. 2d). We further used the three-chamber test to probe animals for their voluntary initiation of social interaction and their preference for social novelty^33^,^34^. In the sociability phase, Ctrl mice spent significantly more time in the chamber with the social partner (S1) than the empty cage (E). In contrast, cKO mice showed no apparent preference (Fig. 2e). In the social novelty phase, a novel partner (S2) was introduced into the previously empty cage. Control mice displayed a preference for S2, while cKO mice spent more time with S1 (Fig. 2f). These abnormal social behaviours were not due to impaired olfactory function as revealed by comparable performance of the Ctrl and cKO mice in the buried food pellet test (Supplementary Fig. 2f).

To determine whether cKOs have deficits in spatial working memory and hippocampus-dependent learning and memory, we subjected the mice to the Y maze, contextual fear conditioning and Morris water maze tests that are often used to study associative learning and spatial learning and memory^24^,^35^. In the Y maze test, cKO mice showed a reduction in spontaneous alternation (Fig. 2g), but a similar number of total arm entries (Supplementary Fig. 2g). In the contextual fear conditioning test, freezing responses were also found to be significantly reduced in cKO mice (Fig. 2h), although this result must be interpreted with some caution in light of differential basal activity observed in locomotor assays. In the Morris water maze, cKO mice took a longer time to reach the hidden platform in the training trials and spent less time in the target quadrant during probe trials, indicating impaired hippocampus-dependent learning and memory (Fig. 2i,j).

Taken together, results from this battery of tests indicate that cKO mice exhibit behavioural deficits in locomotor activity, sensorimotor gating, social behaviour and spatial learning and memory.

### Perturbation of hippocampal synaptic function

**Figure 3:**
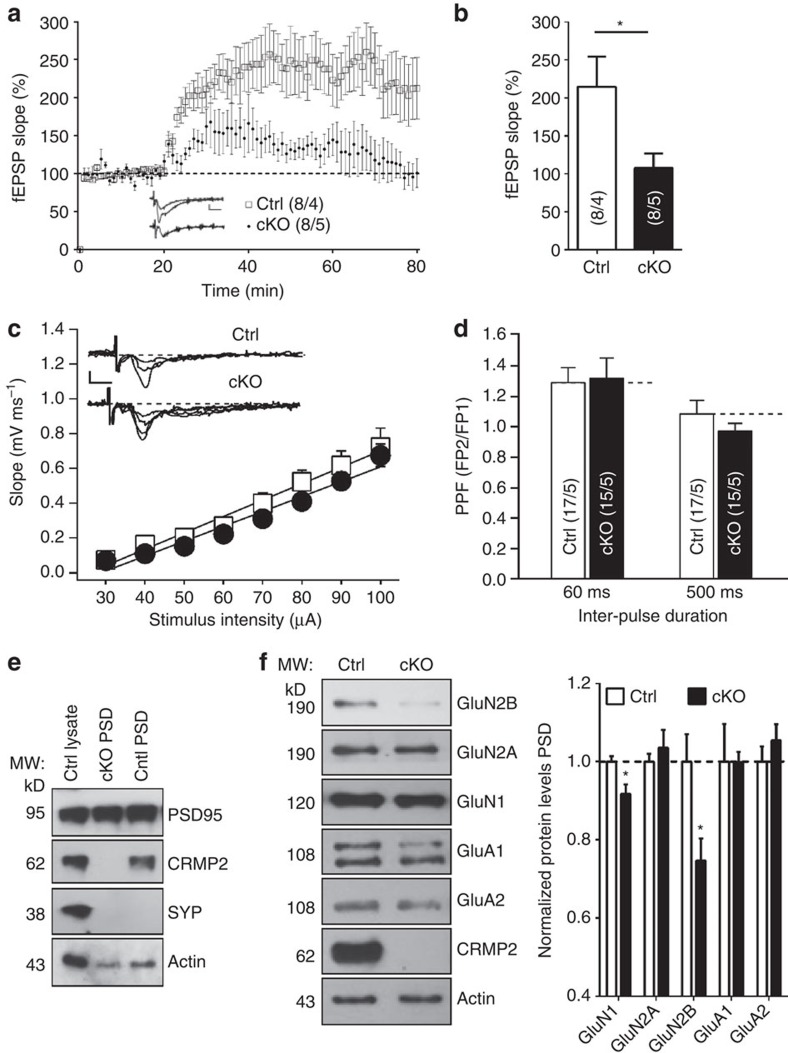
Brain-specific *Crmp2* cKO mice exhibit hippocampal synaptic dysfunction. (**a**) cKO hippocampal slices show impaired TBS-induced LTP in acute brain slices of 8-week-old mice. (**b**) Summary of LTP amplitude in the last 10 min. (**c**) cKOs show normal basal synaptic transmission as reflected by the input–output curve. (**d**) The paired-pulse facilitation ratio at intervals of 60 and 500 ms in Ctrls and cKOs. (**e**) CRMP2 is expressed in PSD fraction of Ctrls, but not present in the PSD fraction from cKO hippocampus as analysed by western blotting. (**f**) Protein levels of glutamate receptor subunits GluN1 and GluN2B are reduced in the hippocampal PSD fractions from cKOs. Five micrograms of protein was loaded in each lane with β-actin as loading control and normalized to the Ctrl levels in the right panel. Data in **e**,**f** are representative of three independent experiments. Values represent mean±s.e.m. (*P<0.05; unpaired *t* test in **b**; others, one-way ANOVA with Tukey *post hoc* test). Scale bar, 1 mV, 5 ms (**a**,**c**).

To elucidate potential consequences of *Crmp2* disruption on synaptic function, we performed electrophysiological recordings of the Schaffer collateral CA1 circuit in brain slices acutely prepared from Ctrl and cKO animals. Theta-burst stimulation (TBS)-induced long-term potentiation (LTP) was substantially reduced in cKO mice (Fig. 3a,b). There was no difference in the input–output curve and the paired-pulse ratio, thus the presynaptic function was less likely to be affected (Fig. 3c,d).

Impaired LTP is most likely due to postsynaptic deficits in synaptic function and/or a reduction in the number of functional synapses^36^. We therefore isolated the hippocampal postsynaptic density (PSD) from both Ctrl and cKO mice to quantify synaptic proteins. We first verified the purity of PSD by western blot with the presynaptic marker SYP and postsynaptic marker PSD-95 (Fig. 3e). The presence of CRMP2 was also confirmed in the PSD fraction from the Ctrl, but not cKO mice (Fig. 3e,f). We then analysed the composition of both NMDA and AMPA receptor subunits. Interestingly, we observed a significant reduction of NMDA receptor subunits, including both GluN2B and GluN1 levels, in the PSD fraction in cKOs (Fig. 3f). These results indicate that defects in synaptic plasticity are likely due to dysregulation of NMDA receptor subunit expression.

### Morphological abnormalities of hippocampal pyramidal neurons

**Figure 4:**
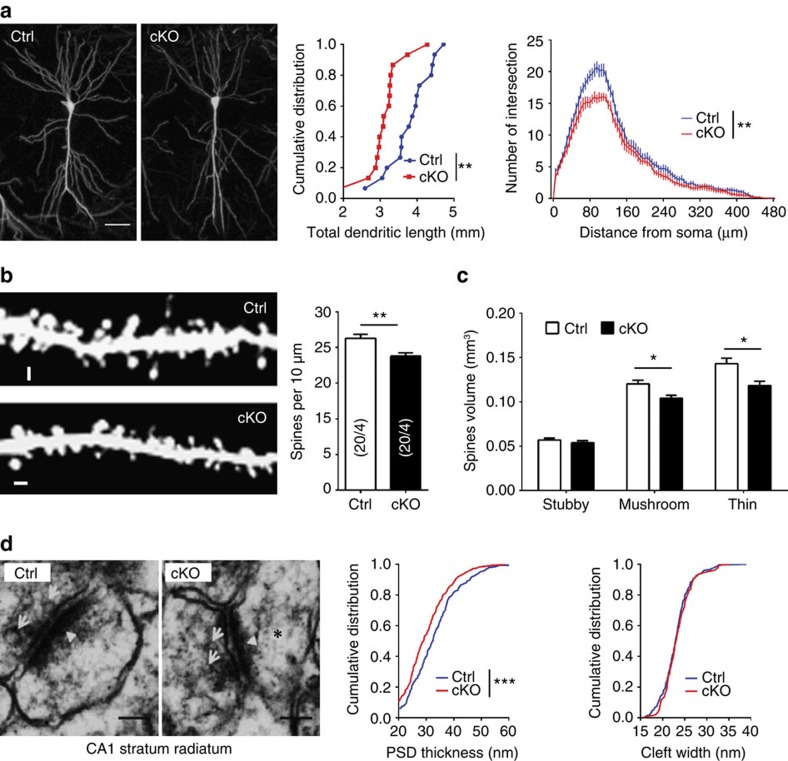
Morphological and ultra-structural neuronal defects in cKO mice. (**a**) Representative confocal projection images of confocal stacks of Thy1-GFPm-labelled pyramidal neurons from the CA1 region of hippocampus. Shown on the right are summary plots of total dendritic length and dendritic complexity by Sholl analysis. Scale bar, 50 μm. (**b**) Representative confocal projection images of dendritic spines from Thy1-GFPm-labelled secondary dendrites of pyramidal neurons in the CA1 region of hippocampus. Shown on right is a summary plot of dendritic spine density. Scale bar, 1 μm. (**c**) Summary plots of spine volume. Note that cKOs have decreased mushroom spines and thin spine volume. (**d**) Representative images of electron micrographs showing the synaptic contacts with presynaptic vesicles (arrows), postsynaptic densities (arrow heads) and dendritic spines (star). Scale bar, 100 nm. Shown on right are quantifications of PSD thickness and cleft width. Values represent mean±s.e.m. (*P<0.05, **P<0.01, ***P<0.001; (**a**,**d**) Kolmogorov–Smirnov test; (**b**) unpaired *t* test; (**c**) one-way ANOVA with Tukey *post hoc* test). Number of animals, neurons and spines analysed in each test are indicated in each bar graph.

The perturbation of hippocampal synaptic plasticity and the aberrant composition of NMDA receptors led us to postulate that those defects might be caused by abnormal neuronal development^6^,^37^. To test this hypothesis, we crossed our *Crmp2* cKO mice with *Thy1-GFPm* transgenic mice. Thy1-GFPm-labelled CA1 pyramidal neurons from cKOs exhibited a significant decrease in both total dendritic length and dendritic arborization complexity (Fig. 4a). Furthermore, there was a significant decrease in the spine density of CA1 pyramidal neurons of cKOs (Fig. 4b). Detailed analysis of different types of spines showed that the volume of mushroom spines and thin spines, but not stubby spines, in apical dendrites was significantly decreased (Fig. 4c). We further examined synapse structures in detail using electron microscopy. Analysis of PSD morphology in hippocampal stratum radiatum showed an apparent decrease in PSD thickness in cKOs, while the synaptic cleft was largely normal (Fig. 4d). These data, together with the reduction in postsynaptic receptor proteins (Fig. 3f), indicate an essential role for CRMP2 in the development and/or maintenance of dendrites and synapses in CA1 pyramidal neurons in the hippocampus.

### Involvement of CRMP2 in adult hippocampal neurogenesis

**Figure 5:**
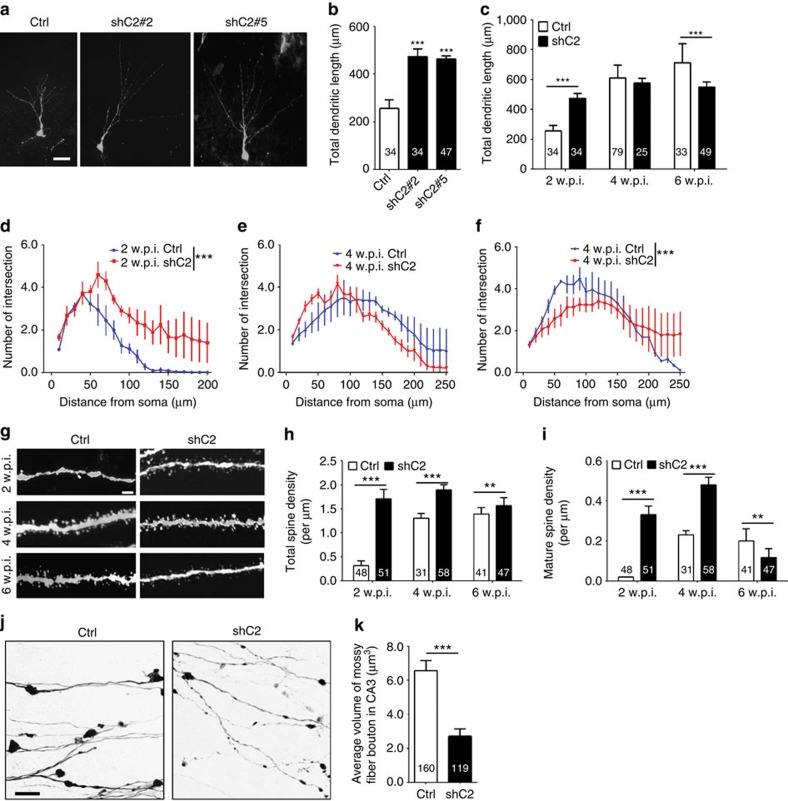
Abnormal dendritic and synaptic development of newborn neurons with CRMP2 deficiency in the adult dentate gyrus. (**a**) Confocal projection images of GFP^+^ neurons at 2 weeks post injection (w.p.i.) of retroviruses co-expressing GFP and control shRNA (Ctrl) or Crmp2 shRNA (shC2#2 and shC2#5). Scale bar, 20 μm. (**b**) Summary of total dendritic length of GFP^+^ newborn neurons expressing different shRNAs. Data were collected from 5, 3, 3 animals for cntl, shC2#2 and shC2#5, respectively. (**c**) Total dendritic length of GFP^+^ neurons at 2, 4 and 6 w.p.i. of retroviruses co-expressing GFP and either Ctrl or *Crmp2* shRNA (shC2) in adult dentate gyrus. Data were collected from 5 (cntl)/3 (shC2), 3 (cntl)/5 (shC2), 3 (cntl)/4 (shC2) animals for 2, 4 and 6 w.p.i., respectively. (**d**–**f**) Sholl analysis at 2 (**d**), 4 (**e**) and 6 w.p.i. (**f**) with the same set of GFP^+^ neurons analysed in **c**. (**g**) Representative confocal projection images of dendritic spines from GFP^+^ neurons at 2, 4 and 6 w.p.i. Scale bar, 2 μm. Data were collected from 3 (cntl)/3 (shC2), 4 (cntl)/5 (shC2), 3 (cntl)/4 (shC2) animals for 2, 4 and 6 w.p.i., respectively. (**h**,**i**) Summary of total spine density (**h**) and mature spine density (**i**), with the same set of GFP^+^ neurons analysed in **g**. (**j**) Representative confocal projection images of mossy fibre boutons. Scale bar, 10 μm. (**k**) Summary of average volume of boutons. Data were collected from 5 cntl and 3 shC2 animals, respectively. Numbers associated with each bar graph refer to the total number of cells analysed under each condition. Values represent mean±s.e.m. (**P<0.01; ***P<0.001; (**b**,**c**,**h**,**i**) one-way ANOVA with Tukey *post hoc* test, (**d**–**f**) Kolmogorov–Smirnov test, (**k**) unpaired *t*-test).

Adult neurogenesis has been suggested to play an important role in brain function and abnormal adult neurogenesis in the hippocampus has been implicated in several neurological and psychiatric disorders^38^,^39^,^40^,^41^,^42^. We therefore characterized the role of CRMP2 in the development of newborn neurons in the adult dentate gyrus. Retrovirus mediated birth-dating and genetic manipulation was used to knockdown *crmp2* expression specifically in newborn cells^43^. We generated multiple shRNAs against mouse *crmp2* and found two to be effective (shC2#2 and shC2#5, Supplementary Fig. 3a). Retrovirus co-expressing GFP and control shRNA or shC2 was stereotaxically injected into the dentate gyrus and newborn granule cells were analysed at 2, 4 and 6 weeks post-retroviral injection (w.p.i.). Unexpectedly, newborn granule cells with CRMP2 knockdown showed markedly enhanced total dendritic length and dendritic complexity compared with that of controls at 2 w.p.i. (Fig. 5a–d). The specificity of shRNAs was further validated by rescue experiments by wild-type CRMP2 expression *in vivo* (Supplementary Fig. 3b). Interestingly, while control newborn neurons exhibited normal development with an increase in total dendritic length and complexity over time at 4 and 6 w.p.i., the CRMP2-deficient granule cells failed to develop further, resulting in reduced total dendritic length and dendritic complexity of more mature adult-born dentate granule neurons (Fig. 5c–f), which is consistent with morphological deficits observed in Thy1-GFPm-labelled mature CA1 pyramidal neurons from cKOs (Fig. 4a).

Newborn neurons are synaptically integrated into the existing circuitry between 2 and 6 w.p.i. (ref. 43). We next examined the synaptic development of newborn granule cells during adult neurogenesis. Surprisingly, CRMP2 deficiency led to an increased number of morphological dendritic spines at all time points examined (Fig. 5g,h). However, the mature mushroom spines were not maintained and the density was significantly lower than that of controls at 6 w.p.i. (Fig. 5i), consistent with our findings that there is a reduction of mature spines in mature CA1 pyramidal neurons of cKO mice (Fig. 4b). We also examined the synaptic boutons of mossy fibers, the synaptic output of dentate granule cells^44^ (Fig. 5j). The CRMP2-deficient neurons exhibited a significantly decreased volume of mossy fibre boutons at 6 w.p.i. (Fig. 5k). Furthermore, there were axonal outgrowth and targeting defects in newborn granule cells with CRMP2 knockdown (Supplementary Fig. 3c). Together, these results suggest an important role of CRMP2 in the morphological development, synaptic maturation and integration of newborn granule cells in the adult dentate gyrus.

## Discussion

Proper brain function relies on stereotyped patterns of neural development. Dysregulation of any process during neural development can have severe and long-lasting consequences. There has been considerable progress in the identification of genetic risk factors for complex psychiatric disorders that have developmental origins, due to the decreased cost of sequencing, increasing numbers of whole-genome sequencing studies, and data analyses from large-scale consortiums^10^,^45^,^46^. How these genetic variants contribute to abnormal neural circuitry formation and its behavioural consequences remain largely unclear. One of the key challenges is to translate the wealth of new information on genetic risk factors into general principles that can guide hypothesis-driven investigations of the aetiology.

Genome-wide linkage studies have consistently identified 8p21 as a susceptibility locus for schizophrenia and the *CRMP2* gene within this locus has been suggested as a risk factor^15^,^20^. Interestingly, altered CRMP2 protein levels have been observed in post-mortem brains of schizophrenia patients, and antipsychotic drugs can modify the level of CRMP2. The physiological role of CRMP2 and its underlying mechanisms remains unknown. Animal models are essential to investigate how individual genes can modulate intact neural circuitry and modify the behaviour of an organism. In this study, we used animal models with a brain-specific knockout, or cell-type-specific knockdown, of *Crmp2*, and provide evidence to support a critical role of *Crmp*2 dysfunction in the expression of several structural abnormalities and behavioural impairments. A growing body of evidence suggests that NMDA receptors play an important role in circuit assembly, and their subunit composition is differentially regulated during postnatal development resulting in unique synaptic properties^47^,^48^,^49^,^50^,^51^,^52^,^53^. In particular, GluN2B-containing NMDA receptors were found to promote the maturation of excitatory synapses in hippocampal neurons during postnatal development^49^. CRMP2 has been shown to bind GluN2B-containing NMDA receptors from mass spectrometry analysis in rat hippocampus and to regulate the surface expression and trafficking of GluN2B, which is necessary for LTP^54^. Consistent with these data, we found that CRMP2 protein is highly enriched in the PSD fraction and required for the maintenance of normal NMDA receptor subunit composition. Importantly, a loss of CRMP2 leads to a significant reduction of GluN2B in the hippocampal PSD fractions in the cKOs. Together with the ultra-structural finding of reduced thickness of PSD in pyramidal neurons from the CA1 region of the hippocampus, our data suggest a potential role of CRMP2-mediated synaptic GluN2B in hippocampal plasticity. Indeed, we found that there is a considerable decrease in theta-burst-induced LTP in the Schaffer collateral circuitry in the cKO. Taken together, we reveal a postsynaptic mechanism underlying CRMP2 regulation of neural plasticity in the hippocampus, which may contribute to spatial learning and memory deficits observed in cKO animals. Additional mechanistic studies are warranted to elucidate the function of CRMP2 in GluN2B trafficking and its contribution to hippocampal-dependent cognition and affection.

The diametric effect of CRMP2 on the morphological development of adult-born granule cells is intriguing. During early developmental stages (2 w.p.i.), the loss of CRMP2 results in increased complexity of dendritic structures and a higher density of immature dendritic spines. At later stages (6 w.p.i.), however, this accelerated growth cannot be maintained, leading to decreases in both the dendritic complexity and mature spine density. These results suggest that CRMP2 also plays an important role in morphological and synaptic development and synapse maintenance. Consistent with this finding, in the *Crmp*2 cKO mice, CA1 neurons exhibited immature characteristics, including a reduced number of mature spines and less dendritic complexity than those of control mice, reflecting a stable phenotype in mature neurons resulting from the loss of CRMP2 during development. Interestingly, an immature dentate gyrus has been associated with schizophrenia based on observations in human patients with schizophrenia and bipolar disorder^39^,^55^. Although this could be due to an arrested state of development, our data support the alternative possibility that this immaturity might be a consequence of failed maintenance of mature synapses, or over-pruning. It would be valuable for future studies to investigate the protracted and dynamic neurogenesis that occurs during adolescence and early adulthood, critical time periods when genetic and environmental factors might interact to contribute to the manifestation of psychiatric disorders such as schizophrenia.

We and others have shown that neuronal polarity and migration were affected by *Crmp2* knockdown using *in utero* electroporation to manipulate the expression of CRMP2 in a subset of cells during early embryonic development^24^,^25^. The cKO brains, however, exhibit largely normal exterior surface features and cortical lamination. This discrepancy could be due to differences in the level and timing of CRMP2 manipulation or potential off-target effect of shRNAs^25^,^56^. While in knockout mice there is sufficient time to allow developmental redundancy mechanisms to evolve^57^, acute gene manipulation by *in utero* electroporation in a subset of cells is less likely to activate these compensatory mechanisms^58^. Significantly enlarged lateral ventricles in the cKO mice are reminiscent of structural brain features in patients with schizophrenia. The exact cause of this structural change is still unknown, but is generally believed to be associated with a reduction of the grey matter, which we did not find in the cKO brain. But this does not rule out the possibility of reduced neural stem cell proliferation, which has also been reported in schizophrenia patients^59^ and in some animal models^60^.

Together, our results suggest a critical contribution of *Crmp2* deficiency in distinct physiological, neuroanatomical and behavioural phenotypes. We observed specific abnormalities associated with neuronal development in the hippocampus that, if recapitulated throughout the brain, could help explain dysregulation of neural circuits and effects on disparate types of behaviours. Our *Crmp2* cKO mice exhibit key structural deficits in neurons that would affect synaptic plasticity, and behavioural impairments analogous to multiple symptomatic domains in schizophrenia. In light of the emerging focus on research domain criteria in the clinical field, it will be important to map genetic risk to functional domains at the cellular, systems and behavioural levels to identify points of convergence and divergence in biological pathways associated with aetiology of psychiatric disease.

Not all genetic risk factors for a single disorder will lead to the same phenotypes. Conversely, a single genetic risk factor is often associated with more than one psychiatric disorder. Hub genes that appear to link many risk genes into the same biological pathway, or core genes that are implicated in multiple cellular and behavioural phenotypes, may be particularly informative in establishing connections among gene networks based on cellular phenotypes, changes in neural structure, behavioural effects, and association with particular psychiatric disorders. *Crmp2* is unusual among the relatively few risk genes that have been studied so far in that we have identified CRMP2-mediated phenotypes from synaptic function to morphological development to gross brain structure. These mice may therefore serve as a valuable new animal model for psychiatric disorder research, amenable to targeted investigations of mechanistic hypotheses of neurodevelopmental dysregulation.

## Methods

### Animals

All the mice were reared on a 12/12 light/dark cycle. All experimental procedures involved were performed according to protocols approved by the Institutional Animal Care and Use Committee at Institute of Genetics and Developmental Biology (IGDB), Chinese Academy of Sciences and Johns Hopkins University School of Medicine. See the following for the genetic background, source, age and sex of mice used for the each experiment.

### Generation of *Crmp2* conditional knockout mice

The mouse *Crmp2* genomic DNA (7.4 kb) was obtained from a C57BL/6 BAC clone (RPC123-414A17). The targeting vector construct was designed to flank exon 3 of the mouse *Crmp*2 gene with two loxP sites to allow Cre-mediated deletion of exon 3 and resulting in a frame shift. The vector was linearized before electroporation into B6/129-derived hybrid ES cells. Selected and confirmed clones were injected into blastocysts from C57BL/6 to generate chimeric mice. Chimeric males were crossed with C57BL/6 to generate heterozygous floxed *Crmp2* (*Crmp2* loxP/+) mice, and homozygous mice (*Crmp2* loxP/loxP) were produced by mating heterozygous mice. Mice homozygous for the floxed *Crmp2* allele were crossed with mice carrying a Nestin promoter-driven Cre transgene (Jackson Laboratory, B6.Cg (SJL)-TgN(Nes-Cre)1Kln), which expresses Cre primarily in the central and peripheral nervous system under the control of the rat nestin promoter and enhancer. The resulting heterozygous mice were used to generate homozygous cKO (*Crmp2* loxP/loxP; Nes-Cre/+, cKO) or heterozygous (*Crmp2* loxp/+; Nes-Cre/+) or homozygous (*Crmp2* loxP/loxP) mice. The homozygous and homozygous conditional knockout mice were used to investigate defects related to CRMP2 deficiency. To obtain GFP labelled neurons, the *Crmp2* cKO mice were crossed with Thy1-GFPm mice (Gift from Yi Zuo, Tsinghua University). The primers used for genotyping were designed for discriminating loxP (forward: 5′-TCTCTATCCACTCTACCACCTC-3′, reverse: 5′- ATTCAATGCCAAGTTATCAGA-3′), Cre (forward: 5′ -CGATGCAACGAGTGATGAGG-3′, reverse: 5′- GCATTGCTGTCACTTGGTCGT-3′) and GFP sequences (forward: 5′- AAGTTCATCTGCACCACCG-3′, reverse: 5′-TCCTTGAAGAAGATGGTGCG-3′).

### Biochemical analyses

Cortex, prefrontal cortex, hippocampus, cerebellum, striatum and hippocampal PSD fractions were prepared from three pairs of 8-week-old Ctrl and cKO male mouse brains as previously described^61^. Adult neural progenitors were isolated from adult mouse hippocampi (C57BL/6) and cultured as previously described^62^,^63^. Protein lysate subjected to western blot analysis were separated on SDS–polyacrylamide gel electrophoresis and probed with specific antibodies; GluN1 (rabbit, 1:1,000; Epitomics, 2824-1,), GluN2A (Cell Signaling Technology, 4205, 1:1,000), GluN2B (Cell Signaling Technology, 4212, 1:1,000), GluA1 (rabbit, 1:1,000; Epitomics, 3861-1), GluA2 (rabbit, 1:1,000; Epitomics, 3520-1), CRMP2 (rabbit, 1:1,000; Cell Signaling Technology, 9393 and Sigma,), p-CRMP2 (rabbit, 1:1,000; Epitomics, 5799-1), SYP (mouse, 1:1,000; Santa Cruz, Sc-17750), PSD-95 (rabbit, 1:1,000; Abcam, ab18258), Actin (mouse, 1:1,000; Cell Signaling Technology, 3700), GAPDH (rabbit, 1:1,000; Cell Signaling Technology, 2118). The relative amount of β-actin or GAPDH was used as loading control. For quantification, the densitometry measurement of each band (Image J) was first normalized to that of Actin or GAPDH and then averaged from at least three independent experiments. Images have been cropped for presentation. Full size images are presented in Supplementary Fig. 4.

### Behavioural tests

All the mice for behavioural tests were housed in groups, 5 mice per cage with mixed genotypes. All behavioural tests were performed during the light phase of the cycle between 09:00 and 17:00. All behavioural testing began on day 8 and mice were allowed 2 h to habituate to the testing rooms before tests. Experimenters were blind to the genotype when behavioural tests were carried out. Male mice at 8–10 weeks of age were used for all the behaviour tests, except for the three-chamber test, in which mice were tested at 5–6 weeks of age. The number of mice per group is indicated in the figures.

### Open-field test

To measure locomotion and spontaneous activity levels, we quantified the behaviour of mice as they freely explored an open-field arena (40 × 40 cm area, 35 cm high walls) using a video tracking system (Smart). Mice were placed in the centre of the arena at the beginning of the session and the total test time was 30 min. To evaluate anxiety-like behaviour, total distance and time spent in the centre region (20 × 20 cm) versus the periphery was compared.

### Home-cage activity

Singly caged mice were habituated in the cage for 12 h and activity was recorded for 10 min. The total distance travelled in the cage was used as a measure of locomotor activity in the cage.

### Three-chamber test for social interaction and social novelty

The three-chamber social test for sociability and response to social novelty was performed as previously described with minor modifications^33^,^64^. Briefly, 5–6-week-old male cKO mice and wild-type (WT) littermate controls (Ctrls) were used in all tests. Age-matched WT male mice were used as Strangers 1 and 2. Stranger mice were habituated to the apparatus by being placed inside wire cages for 25 min each day for 3 days before the test. The three-chamber apparatus consisted of a transparent acrylic box with removable floor and partitions dividing the box into three equally sized chambers (19 × 40 cm). Each chamber could be closed and opened with a door. For the sociability test, the test mouse was put in the middle chamber and left to habituate for 5 min at which time Stranger 1 was introduced into a wire cage in the left side chamber and an empty wire cage on the right side chamber. The dividers were then raised and the test mice were allowed explore freely all three chambers for a 5 min session. Following the 5 min session, the animal was allowed for another 5 min (post-test) to become familiar with the Stranger 1 mouse. Following this, Stranger 2 was introduced into the previously empty wire cage and again the test mouse was allowed to freely explore all three chambers for a 5 min session. Time spent in each chamber and the test mouse’s trajectory were recorded using Avta Maze software (Anilab Software & Instruments).

### Nest building assay

The nest building test was performed as previously described with minor modifications^65^. Briefly, one square piece of material made of cotton fibre (9 × 9 cm) was put in a cage with an individual mouse. Pictures of the nests were taken 16 h later. The quality of the nest was assessed using the following score: 1, nest not noticeably touched; 2, nest partially torn up; 3, mostly shredded but not identifiable nest site; 4, an identifiable but flat nest; 5, a well-defined nest with walls.

### Startle response and prepulse inhibition

The startle response and PPI test was performed as previously described^66^ (Med Associates). Mice were acclimated to the test room for 1 h before the test. The test began with a 5 min acclimation period when the mice were left in the chamber’s cylinder undisturbed. The remainder of the test session consisted of three blocks of trials. The first block consisted of five 40 ms, 120 dB sound bursts used as startle stimuli, presented with varying intertrial intervals (10–15 s). The second block consisted of 12 trials each, with four different trial types: startle only, or a 20 ms prepulse sound at 4, 8 or 12 dB above the background noise level (69 dB) presented 120 ms before the startle stimulus. The third block consisted of five trials each, with different startle stimuli: 90, 100, 110 or 120 dB. The trial types were presented in pseudorandom order throughout each block with an average intertrial interval of 15 s (range from 10 to 20 s). For PPI, the average startle response was used as the primary dependent measure of the startle reflex. Percentage of prepulse inhibition was calculated using the following formula: (100−(prepulse+startle/startle alone) × 100), where the startle alone mean response was obtained from trials conducted in the absence of prepulse stimuli.

### Contextual fear conditioning

The conditioning apparatus consisted of a shock-chamber set up in a sound attenuated box (Panlab). On the training day, mice were placed within the conditioning chamber for 2 min of habituation before three 2 s, 0.7 mA footshocks were delivered at 58 s intervals and then returned to home cages. Mice were tested 24 h later for 5 min in the same conditioning chamber. Freezing was defined as lack of motion, except for respiration and scored by an automated system (SMART).

### Morris water maze

The Morris water maze was used to measure acquisition and expression of spatial memory^67^. A circular water tank (120 cm diameter and 40 cm height) was filled with water (22 °C, 25 cm deep) and in the presence of a constellation of spatial cues visible to the mice. Nontoxic white powder paint was added to the water to make the surface opaque and to hide the escape platform (circular platform, 6 cm in diameter, 1 cm below the surface). The experimental protocol required five days of acquisition with the platform in place (four trials per day) and removal of the platform on the sixth day for a probe test. During the acquisition phase, the platform stayed in the same location for each animal. At the beginning of each trial, the mouse was placed into one of the four quadrants facing the wall and the starting location varied pseudorandomly across trials for each mouse. Mice were given 60 s to find the platform, at which point the experimenter would guide the animal to the platform if necessary. Mice remained on the platform for 30 s, and were then dried with a towel and placed under a 37 °C lamp between trials. To measure the rate of acquisition, the latency to reach the platform was averaged over all four trials each day. For the probe trial, mice were given 60 s to swim and the trajectory and amount of time spent in each quadrant was quantified using a video tracking system (SMART).

### Electron microscopy

Electron microscopy was performed as previously described^64^,^68^. Briefly, mice were assigned a code before dissection to maintain a blinded genotype across all procedures, including dissection, sample processing, imaging and quantification. Dissected hippocampus from fixed brain with 4% paraformaldehyde (PFA, pH7.4) was transferred into a 4% glutaraldehyde solution after 24 h post-fixation (4% PFA) and kept at 4 °C for 3 days. The samples were washed twice, 20 min each, in 7.5% sucrose, 0.1 M sodium cacodylate buffer, then post-fixed in 1% osmium tetroxide for 2 h with initial microwave treatment for 6 min. Next, the samples were washed twice in 0.11 M veronal acetate buffer for 20 min each. Following en-block staining in 1% uranyl acetate in distilled water for 1 h the samples were washed twice in 0.11 M veronal acetate buffer for 20 min each. Samples were dehydrated using serial dilutions of ethanol (70%, 95%, 2 × 100%) for 20 min each, with initial microwave treatment of 2 min. Samples were then treated for 20 min twice with propaline oxide and impregnated with 50:50 propaline oxide: Epon resin overnight at 4 °C, with initial microwave treatment for 3 min. Next, the samples were impregnated with 100% Epon resin, three changes of 2 h each, with initial microwave treatment for 3 min each. Tissue samples were embedded in moulds and incubated for 48 h at 60 °C. Tissues were sectioned to 65 nm with a Leica ultra-microtome. Grids were viewed without staining directly on an electron microscope (JEOL; JEM 1400) at 80 kV and digital images were captured with CCD camera. PSD and synaptic cleft measurements were performed using Image J (NIH).

### Field electrophysiological analysis of acute hippocampal slices

We performed LTP analyses of the Schaeffer collateral to CA1 circuit in 400 μm hippocampal slices prepared from 8-week-old mice. Field electrophysiological recordings were performed as previously described^69^. Briefly, the mice were anaesthetized with pentobarbital, and we quickly cooled the brain with dissection solution which contained (in mM): 0.5 CaCl_2_, 3 KCl, 26 NaHCO_3_, 5 MgCl_2_, 1 NaH_2_PO_4_, 10 glucose, 213 sucrose, PH 7.3–7.4, 290–300 mOsM. The hippocampal slices were incubated in ACSF (containing (in mM): 125 NaCl, 10 glucose, 26 NaHCO_3_, 5 KCl, 2.6 CaCl_2_, 2 NaH_2_PO_4_, 1.3 MgCl_2_, PH 7.3–7.4,290–300 mOsM) at room temperature for 1 h with 95% O_2_/5% CO_2_. For recording, the slice was removed to a recording chamber, which superfused with 30 °C ACSF. Stimulation (0.5 ms) pulses were delivered using a concentric bipolar electrode, and the EPSPs were recorded by a glass pipette (4–7 MΩ) filled with ACSF. The LTP was induced by a TBS protocol, after recording a 20 min baseline. All electrophysiological analysis was performed by experimenters blinded to the genotypes of the mice.

### Constructs

Engineered self-inactivating murine onco-retroviruses were used to co-express shRNAs under the U6 promoter and GFP or RFP under the Ubiquitin promoter (pSUbGW/pSUbRW vector), or to co-express mouse CRMP2 (without the 3′ UTR) under the Ubiquitin promoter and GFP following the IRES sequence (pCUXIE vector), specifically in proliferating cells and their progeny *in vivo*. shRNAs against C*rmp2* were designed to target the mouse *Crmp2* gene with following sequences: shRNA-CRMP2#2 (shC2#2): 5′-GCAGCCAAAGTCTTCAACCTT-3′, and a previously characterized shRNA targeting*Crmp*2, shRNA-CRMP2#5 (shC2#5): 5′-ACTCCTTCCTCGTGTACAT-3′. The control shRNA vector (shRNA-C1; Ctrl) contains a scrambled sequence without homology to any known mammalian mRNA: 5′-TTCTCCGAACGTGTCACGT-3′ (Qiagen).

### *In vivo* genetic manipulation of neural progenitors

Adult female C57BL/6 mice (7–8 weeks old; Charles River) housed under standard conditions were anaesthetized. Concentrated retroviruses were stereotaxically injected into the dentate gyrus at 4 sites (0.5 μl per site at 0.25 μl min^−1^) with the following coordinates (posterior=2 mm from Bregma, lateral=±1.6 mm, ventral=2.7 mm; posterior=3 mm from Bregma, lateral=±2.6 mm, ventral=3.2 mm) as previously described^40^,^70^.

### Dendritic and spine analyses

For analysis of the CA1 region dendrites and spines, Coronal brain sections (100 μm) were prepared from age-matched Ctrl and cKO with Thy1-GFPm. Slides were individually coded and randomly ordered for image acquisition. Images of dendrites (randomly 5 neuron per mouse) were acquired on Zeiss LSM 700 confocal microscope with a 20 × lense. Images of the 2nd segment apical dendrite spine were acquired on LSM 700 confocal microscope with 100 × 1.4 numerical aperture (NA) lense and 2 × optical zoom. The investigator was blind to genotype during image acquisition and analysis of spine morphology. For dendritic analysis, both apical and basal processes of CA1 pyramidal neuron were analysed. For analyses of adult newborn neurons, coronal brain sections (50 μm) were prepared from retrovirus-injected mice and processed for immunostaining using following GFP antibody (goat, 1:500; Rockland & Abcam ab290). Images were acquired on confocal system (Zeiss LSM 700 and 510). Images for dendritic and spine morphology were deconvoluted with Auto Quant X (Media Cybernetics) using the blind algorithm, which employs an iteratively refined theoretical PSF. No further processing was performed prior to image analysis. For visualization, brightness and contrast levels were adjusted using Image J (NIH). For analysis of dendritic development, three-dimensional (3D) reconstructions of entire dendritic processes of each GFP^+^ neuron were obtained from Z-series stacks of confocal images. The two-dimensional (2D) projection images were traced with NIH Image J. All GFP^+^ dentate granule cells with largely intact dendritic trees were analysed for total dendritic length as previously described^43^. The measurements did not include corrections for inclinations of dendritic process and therefore represented projected lengths. For complete 3D reconstruction of spines, consecutive stacks of images were acquired using an excitation wavelength of 488 nm at high magnification (× 63, five times zoom) to capture the full depth of dendritic fragments (20–35 μm long) and spines using a confocal microscope (Zeiss). Confocal image stacks were deconvoluted using a blind deconvolution method (Auto Quant X, Media Cybermetics). The structure of dendritic fragments and spines was traced using 3-D Imaris software using a ‘fire’ heatmap and a 2D *x*–*y* orthoslice plane to aid visualization (Bitplane). Dendritic fragments were traced using automatic filament tracer, whereas dendritic spines were traced by means of an autopath method with the semi-automatic filament tracer (diameter; min: 0.1, max: 2.0, contrast: 0.8). For spine classification, a custom MatLab (MathWorks) script was used based on the algorithm; stubby: length (spine)<1.5 and max width (head)<mean_width (neck) *1.2; mushroom: max width (head) >mean width (neck) *1.2 and max_width (head) >0.3; if the spine was not classified as mushroom or stubby, it was defined as long thin. Axonal bouton volume from axonal fragments was measured using 3D Imaris software using a magic wand menu (Bitplane) after deconvolution.

### Immunostaining

Coronal brain sections (40 μm in thickness) were prepared from fixed brain with 4% paraformaldehyde and immunostaining was as performed as previously described^43^. Antibodies used for the immunostaining were Ctip2 (rat, 1:250; Abcam, ab18465), Satb2 (mouse, 1:250; Abcam, a51502), Tbr1 (rabbit, 1:500; Abcam, ab31940), FoxP1 (rabbit, 1:500; Abcam, ab16645), CRMP2 (rabbit, 1:100; Cell signaling Technology, 9393 & Sigma,), NeuN (mouse, 1:250; Abcam, ab77315), GFP (goat, 1:500; Rockland & Abcam ab290).

### Statistical analysis

All data represent means±s.e.m. For two independent data comparisons, unpaired *t*-test and Kolmogorov–Smirnov tests were used to determine statistical significance. For multiple comparisons, ANOVAs were used as indicated in the text. *P<0.05, **P<0.01, ***P<0.001. All statistical analyses were performed using Origin software (OriginLab), Matlab (Mathworks), Excel 2013 (Microsoft) or GraphPad Prism6.0.

### Data availability

The authors declare that all data supporting the findings of this study are available within the article and its Supplementary Information Files.

## Additional information

**How to cite this article:** Zhang, H. *et al*. Brain-specific *Crmp*2 deletion leads to neuronal development deficits and behavioural impairments in mice. *Nat. Commun.* 7:11773 doi: 10.1038/ncomms11773 (2016).

## Supplementary Material

Supplementary Information Supplementary Figures 1 - 4, Supplementary Materials and Methods and Supplementary References
