# Publisher Correction: Brain-specific *Crmp2* deletion leads to neuronal development deficits and behavioural impairments in mice

**DOI:** 10.1038/ncomms16229

**Published:** 2018-07-18

**Authors:** Hongsheng Zhang, Eunchai Kang, Yaqing Wang, Chaojuan Yang, Hui Yu, Qin Wang, Zheyu Chen, Chen Zhang, Kimberly M. Christian, Hongjun Song, Guo-li Ming, Zhiheng Xu

Nature Communications 7: Article number: 11773; DOI: 10.1038/ncomms11773 (2016); Published 06 01 2016, Updated 07 18 2018

The original HTML version of this Article had an incorrect article number of ‘0’; it should have been ‘11773’. This has now been corrected in the HTML version of the Article. The PDF version was correct from the time of publication.

