# Supplementary material for: Brain-specific Crmp2 deletion leads to neuronal development deficits and behavioural impairments in mice
Source: Nat Commun. 2016 Jun 1;7:11773. doi: 10.1038/ncomms11773 (PMC4895353; doi:10.1038/ncomms11773)
Supplement: Supplementary Information — Supplementary Figures 1 - 4, Supplementary Materials and Methods and Supplementary References [file ncomms11773-s1.pdf]

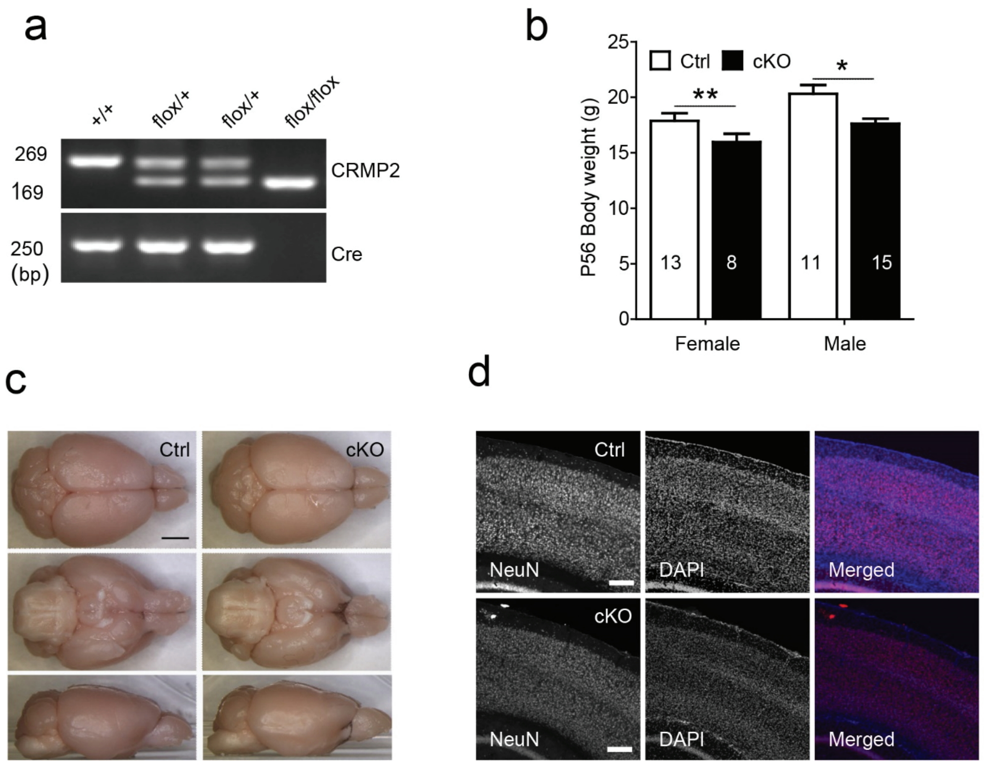


**Supplementary Figure 1. Decreased body weight but normal cortical lamination in *Crmp2* cKO mice.**

(**a**) Example of genotyping result distinguishing wild-type littermates (Ctrls) from heterozygous or homozygous *Crmp2* cKO mice. (**b**) Quantification of whole body weight. cKOs have reduced body weight. Number of animals used are indicated in the figures. Values represent mean ± s.e.m. (**$P < 0.01$; *$P < 0.05$; one way ANOVA with Tukey post-hoc test; Female Ctrl vs cKO: P = 0.0193, $F(1,18) = 6.6001$; Male Ctrl vs cKO: P = 0.0053, $F(1,24) = 9.4053$). (**c**) Representative images of mouse brains from cKOs and Ctrls. Scale bar: 2 mm. (**d**) Representative images of P56 brain sections from cKOs and Ctrls stained with NeuN antibody. Scale bars: 200 μm.

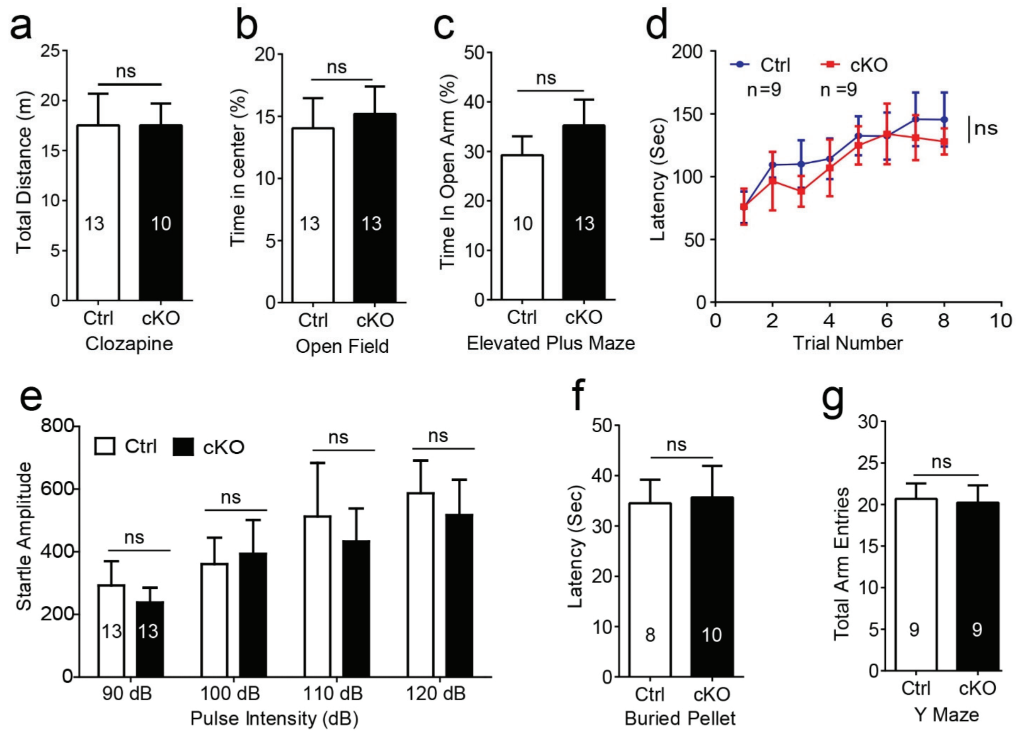


**Supplementary Figure 2. Additional behavioral tests in Ctrl and cKO mice.**
(**a**) No apparent difference between clozapine-treated cKO and Ctrl mice in distance traveled in the open field test, performed 30 min after clozapine administration (P = 0.9999, t(21) = 0.0001351). (**b**) No significant difference between cKOs and Ctrls in the percentage of time spent in the center area during the open field test (P = 0.7283, t(23) = 0.3516). (**c**) cKOs display similar percentage of time spent in the open arm compared to Ctrls in the elevated pulse maze test (P = 0.3631, t(20) = 0.9308). (**d**) cKOs have normal coordination and motor learning ability in the rotarod test (P = 0.9817, F(1,64) = 0.5901). (**e**) cKOs display normal startle response at increased pulse intensity (90 dB Ctrl vs cKO: P = 0.5567, F(1,24) = 0.3552; 100 dB Ctrl vs cKO: P = 0.8121, F(1,24) = 0.0577; 110 dB Ctrl vs cKO: P = 0.6968, F(1,24) = 0.1555; 120 dB Ctrl vs cKO: P = 0.6544, F(1,24) = 0.2055). (**f**) cKOs and Ctrls have similar latency in finding the buried food pellet in the buried food pellet test (P = 0.8884, t(16) = 0.1426). (**g**) cKOs and Ctrls show similar total arm entries in the Y maze test (P = 0.8758, t(16) = 0.1589). Number of animals used in each test are indicated in the figures. All data are means ± s.e.m. (ns: P > 0.05 (**d & e)** ANOVA; all others, unpaired t-test).

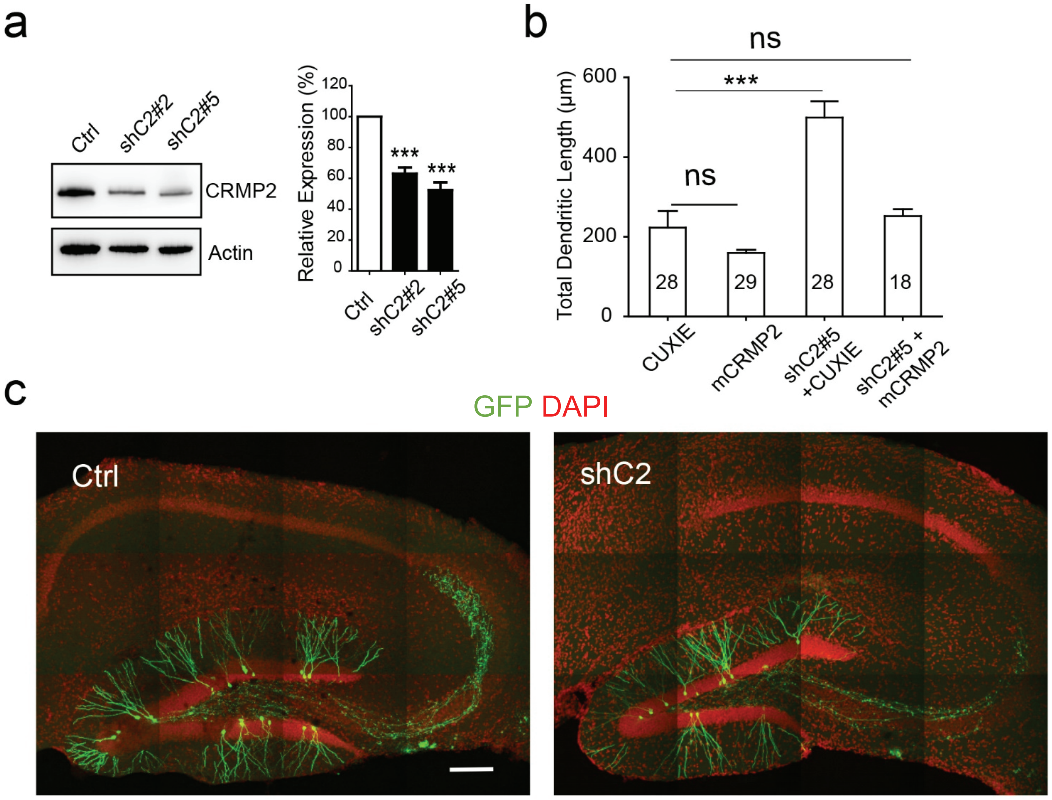


**Supplementary Figure 3. *Crmp2* knockdown in adult-born new dentate granule neurons leads to abnormal dendritic and axonal development.**

(**a**) Validation of *Crmp2* shRNAs (shC2) knockdown efficacy. Retroviruses expressing shRNA and GFP were used to infect cultured mouse adult neural progenitors. Cell lysates were subject to Western Blot analysis for Crmp2 and β-actin. Right panel: quantification. Values represent Mean ± s.e.m. (n = 5; ****P* < 0.001, one way ANOVA with Tukey post-hoc test; Ctrl vs shC2#2: *P* = 1.7658E-5, F(1, 8) = 82.0693; Ctrl vs shC2#5: P = 1.5413E-5, F(1,8) = 85.1547). (**b**) Rescue of enhanced dendritic development in Crmp2 deficiency newborn neurons by Crmp2 expression. Retroviruses co-expressing dsRed and shC2#5 and those co-expressing GFP and mouse Crmp2 (mCrmp2) or GFP alone (CUXIE) were co-injected into the dentate gyrus of adult mice and analyzed at 2 wpi. Numbers associated with each bar graph refer to the number of neurons analyzed from at least two animals under each condition. Values represent mean ± s.e.m. (****P* < 0.001, ns: P > 0.05; one way ANOVA with Tukey post-hoc test; CUXIE vs mCRMP2: P = 0.00598, F(1, 55) = 8.1786; CUXIE vs shC2#5+CUXIE: P = 2.6565E-6, F(1, 54) = 27.5309; CUXIE vs shC2#5 + mCRMP2: P = 0.8415, F(1, 43) = 0.0405)). (**c**) Defective axonal targeting of newborn neurons with Crmp2 deficiency in the adult dentate gyrus. Shown are representative confocal images of $GFP^+$ newborn neurons expressing control shRNA (Ctrl) or Crmp2 shRNA# 2 (shC2) at 2 wpi. Scale bar: 100 μm.

Fig. 1d

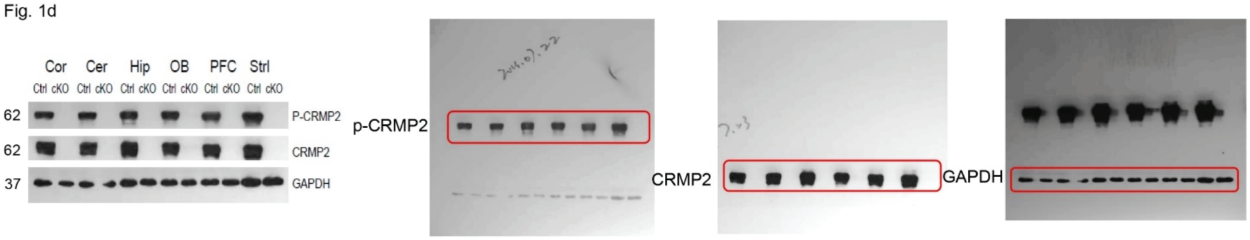


Fig. 3e

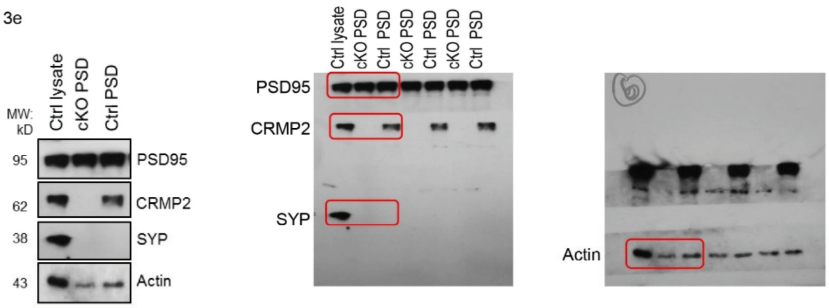


Fig. 3f

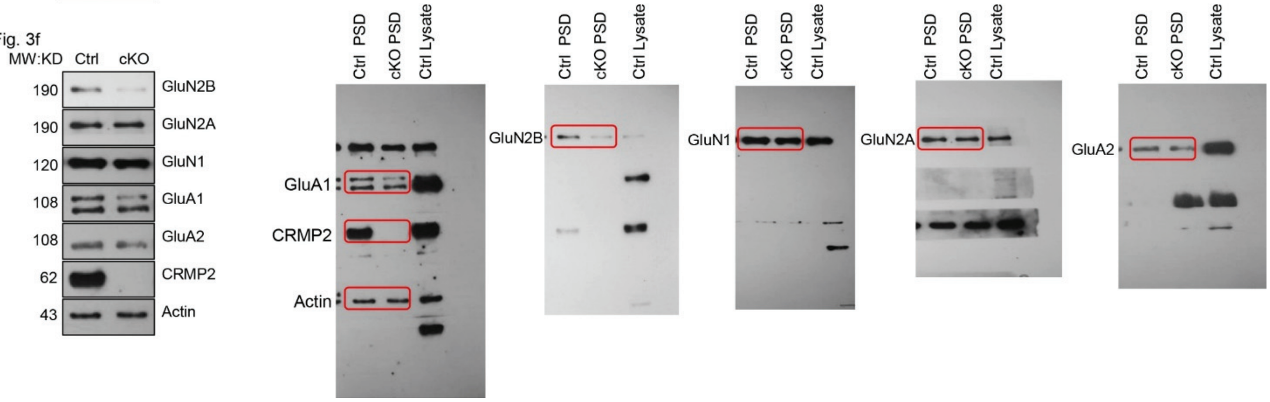


Fig S3a

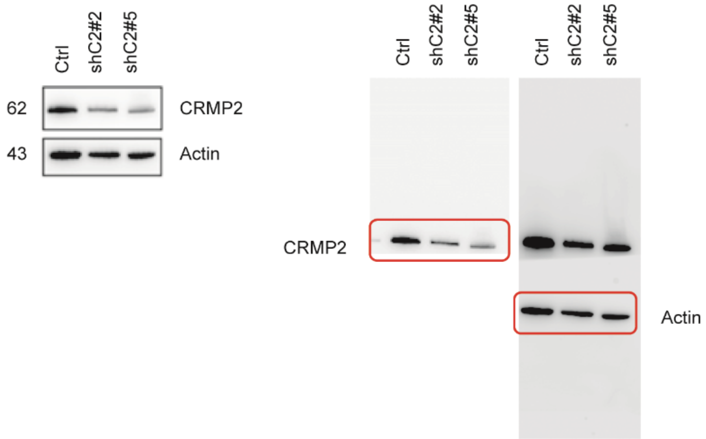


**Supplementary Figure 4. Full images of gels showed in Fig. 1d, 3e, 3f and Fig S3a**

## Supplemental Materials and Methods

### Elevated plus maze

The elevated plus maze was conducted as described previously[1]. Briefly, the apparatus was constructed of black stainless steel and consisted of four arms (30 × 5 cm): two closed arms with 30 cm high black walls and two open arms with raised lips of 0.5 cm. All four arms were connected by a center platform of 5 × 5 cm. The maze was elevated to a height of 50 cm above the ground. Each mouse was placed on the center platform facing an open arm to initiate the test period of 5 min. The video tracking system (Smart) was used to score the time spent in the open arms and the number of entries into the open and closed arms (an arm entry was defined as all 4 paws in an arm).

### Rotarod test

Motor coordination, balance, and motor learning were evaluated with an accelerating rotarod assay[2]. Mice were assessed for their ability to maintain balance on a rotating bar that accelerated from 4 to 40 rpm over a period of 4-min. Latency to fall from the rod was recorded. Each mouse was given four trials per day for 2 days with an interval of 60 min.

### Y maze spontaneous alternation test

The Y maze spontaneous alternation test was performed as previously described[3]. A Y maze with three identical arms of transparent Plexiglas (40 × 4.5 × 12 cm) 120° apart was placed in the test room with clues located in the periphery of the room to allow visual orientation. Each mouse was placed at the end of one arm facing the center and allowed to freely explore the apparatus with the experimenter out of sight. All sessions were video recorded through a camera mounted above the maze and behavior was evaluated using by automatic video tracking (Anilab). Entries into each arm were scored for 8 min. Alternation behavior was defined as consecutive entries into each of the three arms without repetition (that is, ABC, BCA…). We defined the percentage of spontaneous alternation as the actual alternations divided by the possible number of alternations (total arm entries − 2) × 100. Total entries were scored as an index of ambulatory activity in the Y maze and mice with scores below 12 were excluded.

### Buried food pellet test

The buried food pellet test was performed as previously described[4]. Mice were placed on a food restricted diet (0.2g chow per mouse/24 h) starting 2 days prior to testing and during the 3-day experimental period. On each test day, mice received one trial per day. In each trial, a single mouse was placed in a test cage (45cm×24 cm×20 cm) to recover a 1 g food pellet. The food pellet was

buried approximately 0.5 cm below the surface of a 3 cm deep layer of mouse bedding material. The location of food pellet was changed daily. The latency to find the food pellet was defined as the time between when the mouse was placed in the cage and when the mouse uncovered the food pellet and grasped it in its forepaws and/or teeth. Mice were allowed to consume the pellet they found and then returned to their home cage. Mice that did not find the food pellet within 5 min were removed and placed back into home cage. The bedding in the test cage was changed between trials.

## Supplemental References


1 Yu, H. *et al.* Variant brain-derived neurotrophic factor Val66Met polymorphism alters vulnerability to stress and response to antidepressants. *The Journal of neuroscience : the official journal of the Society for Neuroscience* **32**, 4092-4101, doi:10.1523/JNEUROSCI.5048-11.2012 (2012).

2 Shiotsuki, H. *et al.* A rotarod test for evaluation of motor skill learning. *Journal of neuroscience methods* **189**, 180-185, doi:10.1016/j.jneumeth.2010.03.026 (2010).

3 Hughes, R. N. The value of spontaneous alternation behavior (SAB) as a test of retention in pharmacological investigations of memory. *Neuroscience & Biobehavioral Reviews* **28**, 497-505 (2004).

4 Nathan, B. P., Yost, J., Litherland, M. T., Struble, R. G. & Switzer, P. V. Olfactory function in apoE knockout mice. *Behavioural brain research* **150**, 1-7, doi:10.1016/S0166-4328(03)00219-5 (2004).
